# Genome-Wide Identification and Characterization of *CLAVATA3/EMBRYO SURROUNDING REGION (CLE)* Gene Family in Foxtail Millet (*Setaria italica* L.)

**DOI:** 10.3390/genes14112046

**Published:** 2023-11-06

**Authors:** Xuemei Ren, Jinjie Chen, Shuwan Chen, Hui Zhang, Li Li

**Affiliations:** College of Life Science, Shanxi Agricultural University, Jinzhong 030801, China; 19835144897@163.com (J.C.); chensw27999@163.com (S.C.); huizhang@sxau.edu.cn (H.Z.); 18634870414@163.com (L.L.)

**Keywords:** foxtail millet, *CLE* gene family, expression pattern, plant hormone

## Abstract

The *CLAVATA3/EMBRYO-SURROUNDING REGION (CLE)* genes encode signaling peptides that play important roles in various developmental and physiological processes. However, the systematic identification and characterization of *CLE* genes in foxtail millet (*Setaria italica* L.) remain limited. In this study, we identified and characterized 41 *SiCLE* genes in the foxtail millet genome. These genes were distributed across nine chromosomes and classified into four groups, with five pairs resulting from gene duplication events. *SiCLE* genes within the same phylogenetic group shared similar gene structure and motif patterns, while 34 genes were found to be single-exon genes. All SiCLE peptides harbored the conserved C-terminal CLE domain, with highly conserved positions in the CLE core sequences shared among foxtail millet, Arabidopsis, rice, and maize. The *SiCLE* genes contained various *cis*-elements, including five plant hormone-responsive elements. Notably, 34 *SiCLE* genes possessed more than three types of phytohormone-responsive elements on their promoters. Comparative analysis revealed higher collinearity between *CLE* genes in maize and foxtail millet, which may be because they are both C_4_ plants. Tissue-specific expression patterns were observed, with genes within the same group exhibiting similar and specific expression profiles. *SiCLE32* and *SiCLE41*, classified in Group D, displayed relatively high expression levels in all tissues except panicles. Most *SiCLE* genes exhibited low expression levels in young panicles, while *SiCLE6*, *SiCLE24*, *SiCLE25*, and *SiCLE34* showed higher expression in young panicles, with *SiCLE24* down-regulated during later panicle development. Greater numbers of *SiCLE* genes exhibited higher expression in roots, with *SiCLE7*, *SiCLE22*, and *SiCLE36* showing the highest levels and *SiCLE36* significantly down-regulated after abscisic acid (ABA) treatment. Following treatments with ABA, 6-benzylaminopurine (6-BA), and gibberellic acid 3 (GA3), most *SiCLE* genes displayed down-regulation followed by subsequent recovery, while jasmonic acid (JA) and indole-3-acetic acid (IAA) treatments led to upregulation at 30 min in leaves. Moreover, identical hormone treatments elicited different expression patterns of the same genes in leaves and stems. This comprehensive study enhances our understanding of the *SiCLE* gene family and provides a foundation for further investigations into the functions and evolution of *SiCLE* genes in foxtail millet.

## 1. Introduction

Multicellular organisms often rely on intercellular communication to coordinate growth and development, regulate physiological activities, and ensure efficient and orderly operation. In plants, intercellular communication is primarily achieved through hormones, peptide ligands, small RNAs, and even gases [1]. Peptide ligands, specifically small peptides with less than 100 amino acids in length, play diverse roles in plant growth, development, and reproduction, as well as symbiotic interactions and stress responses [2]. The *CLE (CLAVATA3/Endosperm surrounding region-related)* gene family encodes the largest known peptides in plants. Research has demonstrated that each *CLE* gene encodes a precursor peptide containing a highly conserved CLE domain. The CLE conserved motif undergoes proteolytic cleavage from the precursor peptide and then experiences further post-translational modifications, such as hydroxylation and glycosylation, to produce mature CLE peptides [3]. The mature peptide can be secreted into the extracellular space and subsequently perceived by receptors located on the plasma membrane of adjacent cells, thereby mediating intercellular communication [4].

The CLE peptide family exhibits widespread distribution in various plant tissues and organs, displaying tissue-specific expression patterns [5]. Specific CLE peptides have been found to exert influence on plant development in specific tissues [6]. The maintenance of shoot apical meristem (SAM) mainly relies on the receptor kinases mediated CLV-WUS (CLAVATA-WUSCHEL) negative feedback regulatory loop [7,8]. Within this regulatory loop, the transcription factor WUS functions as a central regulatory element in maintaining the activity of stem cells within the SAM [9]. The CLV-WUS negative feedback pathway that maintains the homeostasis of the SAM was first discovered in *Arabidopsis thaliana*. However, this pathway is highly conserved in higher plants. In rice (*Oryza sativa*), the *FON2* (*FLORAL ORGAN NUMBER 2*) and *FCP1* (*FON2*-*like CLE PROTEIN 1*) genes encode homologous proteins of CLV3, both containing conserved CLE domains. However, unlike in Arabidopsis, where *CLV3* simultaneously regulates the SAM both in vegetative and reproductive phases, *FON2* and *FCP1*, respectively, maintain the homeostasis of the SAM during the reproductive and vegetative growth phases [10,11]. The rice gene *FON1*, the maize gene *TD1* (*THICK TASSEL DWARF 1*), and the tomato (*Solanum lycopersicum* L.) gene *FAB* (*FASCIATED AND BRANCHED*) encode homologous proteins of CLV1 [12,13,14]. The maize gene *FEA2* (*FASCIATED EAR 2*) and the tomato gene *SlCLV2* encode homologous proteins of CLV2 [14,15]. Mutations in these genes lead to enlarged SAM and the formation of additional floral organs.

The regulation of plant development by CLE peptides often involves the production and participation of phytohormones, and the interaction between phytohormone signaling and CLE peptides has been widely studied. The *cle42* mutant displayed earlier senescence phenotypes, while overexpression of *CLE42* or application of the synthesized 12-amino-acid peptide (CLE42p) delayed leaf senescence under natural and dark conditions [16]. Further research found that *CLE42* can delay leaf senescence by suppressing ethylene biosynthesis in Arabidopsis. The classical phytohormone cytokinin and the small CLE peptides are potent regulators of cell division and cell differentiation. *CLE* and cytokinin signaling are highly intertwined developmental regulators with antagonistic functions in shoots and synergistic functions in roots [17]. The application of gibberellic acid 4 (GA4) to the shoot apex of the GA-deficient mutant *ga3ox1/ga3ox2* induces substantial accumulation of *CLE6* in the root and rescued the short-root phenotype, which suggests that *CLE6* plays a systemic role in shoot growth under the influence of GA in Arabidopsis [18]. *AtCLE9 is* expressed in stomata and acts as an essential regulator in the induction of stomatal closure, while in abscisic acid (ABA)-deficient mutants, *CLE9*-induced stomatal closure was impaired, indicating that ABA is required for *CLE9*-mediated guard cell signaling [19]. Arabidopsis *CLE5* and *CLE6* are expressed specifically at the base of developing leaves and floral organs, and their transcript levels are regulated by auxin to modulate the final leaf morphology [20]. The mutual cooperation or antagonistic regulation between CLE peptides and plant hormones in various plant tissues is gradually being discovered.

The *CLE* gene family is gradually being discovered in various species. So far, the *CLE* gene family has been identified and analyzed in several species, such as Arabidopsis [1], tomato [21,22,23], cucumber [24], soybean [25], *Populus trichocarpa* [26], grape [27], wheat [28], *Brassica napus* [29], cotton [30], and potato [31]. Foxtail millet is an important C_4_ cereal crop that has outstanding drought resistance and barren resistance. In addition, foxtail millet is a diploid (2n = 2X = 18) self-pollinated crop, which has the advantages of a small genome (about 400 Mb), few repeat sequences, and easy mutagenic and mutant screening, and it is developing into a new model plant for C_4_ grasses [32]. However, no studies related to the *CLE* gene family in foxtail millet have been reported. In this study, we identified 41 *SiCLE* genes by whole-genome analysis in foxtail millet and conducted a phylogenetic tree of the *CLE* genes from four species, including foxtail millet, Arabidopsis, maize, and rice. Focusing on *SiCLE* genes in foxtail millet, we analyze the chromosome location, gene structure and conserved motifs, *cis*-acting elements, and tissue-specific expression pattern based on RNA-seq data. Further, we examine the response of *SiCLE* genes to different plant hormones in foxtail millet. Our study provides a comprehensive understanding of the *SiCLE* gene family that opens the gate to further detailed studies on the gene function and evolution of *SiCLE* genes in foxtail millet.

## 2. Materials and Methods

### 2.1. Genome-Wide Identification of SiCLE Genes in Foxtail Millet

The foxtail millet genome, proteome, coding sequences, and GFF annotation file were downloaded from MDSi, Multi-omics Database for *S. italica* (http://foxtail-millet.biocloud.net/home, accessed on 26 November 2021) [33]. CLE peptide sequences of Arabidopsis (https://www.arabidopsis.org/, accessed on 26 November 2021), maize (https://maizegdb.org/, accessed on 26 November 2021), and rice (https://rapdb.dna.affrc.go.jp/, accessed on 26 November 2021) were downloaded and used as queries for the Basic Local Alignment Sequence Tool for Protein (BLASTP) [34] to search the proteomes of foxtail millet with E-value of 1 × 10^−5^. Then, the Hidden Markov Models (HMMs) of the CLE domain were used to perform HMM searches on the protein sequences of foxtail millet using the HMM-based search program Hmmer3 (http://hmmer.org, accessed on 26 November 2021) [35,36]. The retrieved candidate gene sequences were further filtered through BLASTP screening. Finally, based on important features of the *CLE* gene family, a more thorough screening was conducted to determine the final *SiCLE* genes in foxtail millet. The SiCLE peptide sequences were subjected to protein physical and chemical property analysis, including molecular weight, isoelectric point, and hydrophilicity, using the ExPASy Proteomics (http://www.expasy.org, accessed on 20 March 2022) [37].

### 2.2. Phylogenetic Analysis and Classification of SiCLE Genes in Foxtail Millet

MAFFT (https://mafft.cbrc.jp/alignment/server/, accessed on 21 March 2022) was used to perform multiple sequence alignment of the CLE protein sequences of Arabidopsis, rice, maize, and foxtail millet [38]. The alignment results were imported into MEGA7.0 software, and a neighbor-joining algorithm with 1000 bootstrap replicates was used to construct the phylogenetic tree [39]. The iTOL (https://itol.embl.de, accessed on 21 March 2022) online tools were used to beautify the phylogenetic tree [40].

### 2.3. Gene Structure and Conserved Motif Analysis of SiCLE Genes in Foxtail Millet

GSDS2.0 (http://gsds.cbi.pku.edu.cn, accessed on 28 November 2021 ) was used to visualize the gene structures of the candidate genes [41]. The online tool Multiple Expectation Maximization for Motif Elucidation suite (MEME, https://meme-suite.org/, accessed on 28 November 2021) was used to search for conserved motifs with a maximum parameter value of 5 and the optimum width was 6 to 200 residue width with an E-value < 1 × 10^−10^ was retained [42].

### 2.4. Cis-Regulatory Elements Analysis of SiCLE Genes in Foxtail Millet

Promoter sequences (2000 bp of sequence upstream of the transcription start site) for *SiCLE* genes were extracted by TBtools and scanned for *cis*-regulatory elements on the PlantCARE database (http://bioinformatics.psb.ugent.be/webtools/plantcare/html/, accessed on 30 March 2022) [43]. The analysis results were plotted using R scripts.

### 2.5. Cene Duplication and Collinearity Analysis of the SiCLEs in Foxtail Millet

The chromosomal localization information for each *SiCLE* gene was obtained from the gene annotation files, and the results were visualized using the Graphics function in TBtools [44]. The *SiCLE* genes were analyzed for duplication events using MCScanX with default parameters. The Dual Synteny Plotter tool in TBtools was used to analyze the conserved synteny between foxtail millet and three other species (Arabidopsis, rice, and maize).

### 2.6. Expression Pattern Analysis of SiCLE Genes in Foxtail Millet

Transcriptome data of different tissues (roots, stems, leaves, grains, and panicles) from Jingu21 at various developmental stages were obtained from the MDSi database. Germinated seeds were sampled at 3 days. Plants were sampled at one-tip-two-leaf stage. Leaf 2 was sampled at heading stage. Neck–panicle internodes, flag–leaf, flag–leaf–sheath, stem, leaf 1, leaf sheath 1, and root were sampled during the filling stage. Immature seeds were sampled at early grain filling stage (S1), middle grain filling stage (S2), late grain filling stage (S3), final grain filling stage (S4), and grain maturation stage (S5) [45]. Immature spikelets were sampled at the S2 and S4 stages. Panicle 1 are primary branches, and panicle 2 are tertiary branches at panicle–branch differentiation stage. TPM values were calculated to assess gene expression levels, and a heatmap was generated using R scripts [46]. Young panicle samples were collected from Jingu21 at two distinct formation stages: stage 1 (approximately 1.0–1.5 mm) when branch meristems were specified and stage 2 (approximately 2.5–3.0 mm) when branch meristems were clearly formed. Root samples were collected from 9-day-old Yugu 1 seedlings treated with ABA (2 μM) and untreated control (CK) in a greenhouse under the following conditions: light intensity of 50,000 LX, 16 h daylight at 28 °C, and 8 h darkness at 22 °C. Subsequently, young panicle and root samples were rapidly frozen in liquid nitrogen and stored at −80 °C. Novogene Bioinformatics Technology Co., Ltd. (Beijing, China) was commissioned to construct the RNA libraries. High-throughput sequencing was performed on the Illumina Hiseq platform with three biological replicates.

### 2.7. Response of SiCLE Genes to Phytohormones in Foxtail Millet

The seedlings of Jingu 21 were cultured with Hoagland hydroponic nutrient solution (Beijing Coolaber Technology Co., Ltd., Beijing, China) and grown in greenhouse with light intensity of 50,000 LX for 16 h in the daylight at 28 °C and 8 h in the dark at 22 °C. Seedlings (28 days old) were treated with plant hormones (Beijing Coolaber Technology Co., Ltd., Beijing, China) including 6-benzylaminopurine (6-BA) (100 µM), abscisic acid (ABA) (100 µM), GA3 (100 µM), jasmonic acid (JA) (100 µM), and indole-3-acetic acid (IAA) (100 µM). Samples of leaves and stems were collected at 0 h, 0.5 h, 2 h, 6 h, and 12 h after the treatments. The samples were frozen in liquid nitrogen and stored at −80 °C. Three replicates were performed for each treatment. RNA from different tissues was extracted by Trizol method using Total RNA Extract Reagent and RNA Extraction solution (Beijing Coolaber Technology Co., Ltd., Beijing, China). Reverse transcription was performed using All-in-One First-Strand Synthesis MasterMix (with dsDNase) (BestEnzymes Biotech Co., Ltd., Lianyungang, China) for Real-time Quantitative Polymerase Chain Reaction (qPCR). F488 SYBR qPCR Mix (Universal) (BestEnzymes Biotech Co., Ltd. Lianyungang, China) was used as a fluorescent dye. Primers were designed using Primer 5.0 (Table S1). We used the gene Si9g37480 as an internal control, which was stably expressed at each growth stage in almost all tissues [32]. Each reaction was performed three times, and the 2^−ΔΔCT^ method [47] was used to calculate the relative gene expression levels.

## 3. Results

### 3.1. Identification of the SiCLE Genes in Foxtail Millet

The whole genome was comprehensively analyzed using HMM search and multiple rounds of BLASTP to identify genes with a significance threshold of E < 10^−5^. Candidate genes with a sequence length exceeding 300 amino acids were excluded based on *CLE* gene characteristics [48]. Furthermore, candidate peptide sequences that contained the conserved CLE motif at the C-terminus were retained after motif prediction using MEME [42]. A total of 41 candidate *CLE* genes were successfully identified in the foxtail millet genome, and they were named *SiCLE1*-*SiCLE41* based on their respective chromosomal locations (Table 1). The *CLE* genes are found in diploid Arabidopsis (32 genes) [1], tomato (52 genes) [22], and cucumber (26 genes) [24], and the number of *SiCLE* genes in foxtail millet is between Arabidopsis and tomato. Nevertheless, this number was higher in polyploid species soybean (84 genes) [25], wheat (104 genes) [28], and *B. napus* (116 genes) [29], which may be attributed to complex gene duplication events throughout evolution.

Physicochemical analysis revealed that the 41 SiCLE peptides ranged from 77 to 127 amino acids, with predicted molecular weights ranging from 7.93 to 13.42 kDa and isoelectric points ranging from 5.45 to 12.16 (Table 1). The majority of SiCLE peptides displayed alkaline properties, with only six being acidic (SiCLE1, SiCLE9, SiCLE14, SiCLE20, SiCLE27, and SiCLE36). Signal peptide prediction identified the presence of signal peptides in 36 out of the 41 CLE peptides. Based on the average hydrophobicity index (grand average of hydropathicity, GRAVY) principle, which classifies proteins as biphasic with indices ranging from −0.5 to 0.5, hydrophobic with positive values, and hydrophilic with negative values, all SiCLE proteins were categorized as biphasic proteins. Despite variations in the number of *CLE* genes among different species, the physicochemical properties of the peptides have not significantly changed [48].

### 3.2. Phylogenetic Analysis and Classification of SiCLE Peptides in Foxtail Millet

**Figure 1:**
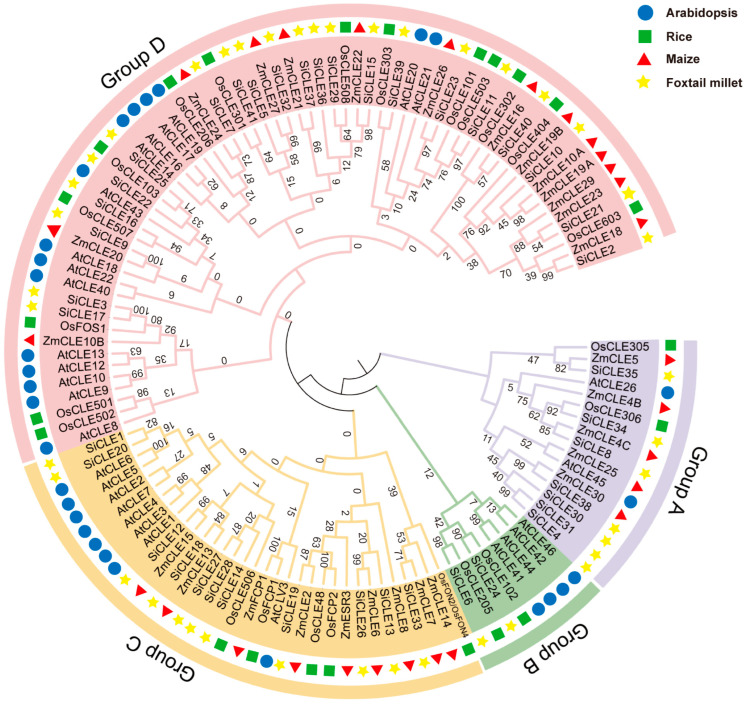
Phylogenetic tree of CLE peptides from foxtail millet, Arabidopsis, rice, and maize. The phylogenetic tree was constructed with the neighbor-joining (NJ) method in MEGA 7.0 software and was divided into four subgroups.

To evaluate the evolutionary relationship of the SiCLE peptides in foxtail millet, Arabidopsis, rice, and maize, a phylogenetic tree using the neighbor-joining (NJ) method was constructed based on the full-length sequences of 41 SiCLE peptides with other CLE peptides identified in the three species (Table S2). All the CLE members clustered into four groups, named Groups A-D, in the phylogenetic analysis (Figure 1). Group C and Group D contain a relatively larger number of CLE genes, with eleven and twenty-one *SiCLE* genes, respectively. Group C CLE peptides include AtCLV3, OsFCP1, OsFCP2, OsFON2/OsFON4, ZmFCP1, and ZmCLE7 [1,10,11,49,50], which all participate in the stem cell differentiation and maintenance in SAM. The eleven *SiCLE* genes in Group C may play a conserved function in the stem cell differentiation and maintenance in the SAM of foxtail millet. Almost all the AtCLE peptides in Group D are called root-active CLE peptides, and they all led to short-root, suppressed protophloem differentiation and consumption of root apical meristem (RAM) phenotypes by applications of individual peptides in vitro [51,52]. The twenty-one *SiCLE* genes may be involved in the stem cell homeostasis in the root of foxtail millet. Group B has the least number of *CLE* genes and contains only two *SiCLE* genes, namely *SiCLE6* and *SiCLE24*. *AtCLE41* and *AtCLE44* belong to Group B. It has been identified that AtCLE41p and AtCLE44p have the ability to inhibit the differentiation of mesophyll cells into xylem cells in an in vitro xylogenic culture system [53]. *SiCLE6* and *SiCLE24* may have similar functions in vascular bundle development in foxtail millet. The remaining seven *SiCLE* genes belong to Group A, and the mechanism of *CLE* genes in this group has not yet been resolved clearly. The results indicate a high degree of conservation and similar functions within each group, suggesting that SiCLE peptides in Groups C and D may play similarly important roles in maintaining homeostasis of the SAM and RAM, respectively. Additionally, Group B may be involved in vascular bundle development.

### 3.3. Gene Structure and Conserved Motif Analysis of SiCLE Peptides in Foxtail Millet

**Figure 2:**
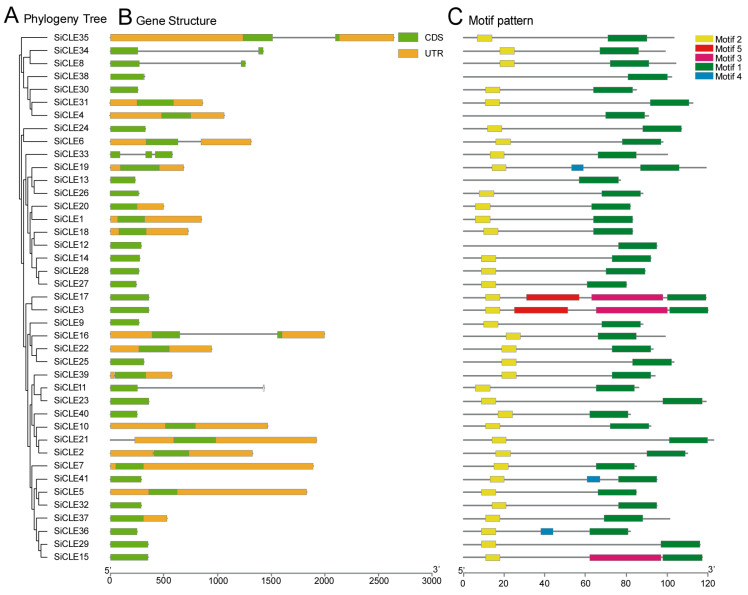
Gene structure and conserved motif analysis of SiCLE peptides. (**A**) Phylogeny tree. (**B**) Gene structure. Coding sequence (CDS) and untranslated region (UTR) are represented by different colored boxes, and introns are represented by lines. (**C**) Motif patterns. Conserved motifs in the SiCLE peptides are represented by different colored boxes. Weblogo plots of the five conserved motifs are shown in Figure S1.

Based on genome annotation, we conducted a comprehensive analysis of the gene structure of *SiCLE* genes (Figure 2). The majority of *SiCLE* genes exhibited either intron-less structures or had only a few introns. Out of the total 41 *SiCLE* genes, 34 were single-exon genes, 6 were two-exon genes, and 1 was a three-exon gene. Moreover, more than half (24/41) of the *SiCLE* genes lacked untranslated regions (UTRs), while the remaining 17 *SiCLE* genes possessed either 3′UTRs or both 5′ and 3′UTRs. The lack of introns and UTRs in SiCLE genes may be attributed to genomic simplification, which minimizes non-coding regions to enhance genome efficiency and replication speed [54]. Notably, *SiCLE* genes within the same phylogenetic cluster demonstrated similar features despite displaying diverse gene structures.

To identify conserved motifs in SiCLE peptides, we utilized the MEME website. A total of five conserved motifs were identified, ranging in length from 7 to 36 amino acids (Figure S1). The CLE precursor peptide is characterized by three main domains: the N-terminal signal peptide region, the central variable domain, and the C-terminal conserved CLE domain, which is highly conserved and approximately 12–13 amino acids in length. The predicted motifs obtained from the MEME online software aligned well with the reported features of CLE precursors [22]. Motif 1, located near the C-terminus, encompassed the typical CLE domain and was present in all 41 members. Motif 2, positioned close to the N-terminus, was found in the majority of members. Motifs 3, 4, and 5 were scattered throughout the middle region of different members, with only a few members containing them.

To investigate the conservation of the CLE core sequences comprising 12–13 amino acids within Motif 1, we performed a comparative analysis among foxtail millet, Arabidopsis, rice, and maize (Figure S2). The results revealed a high degree of similarity between foxtail millet and the other three species. Specifically, positions 2, 4, 5, 7, 8, 10, and 12 exhibited notable conservation, indicating their significance in CLE peptide maturation and functionality [26,55].

### 3.4. Cis-Regulatory Elements in SiCLE Gene Promoters

**Figure 3:**
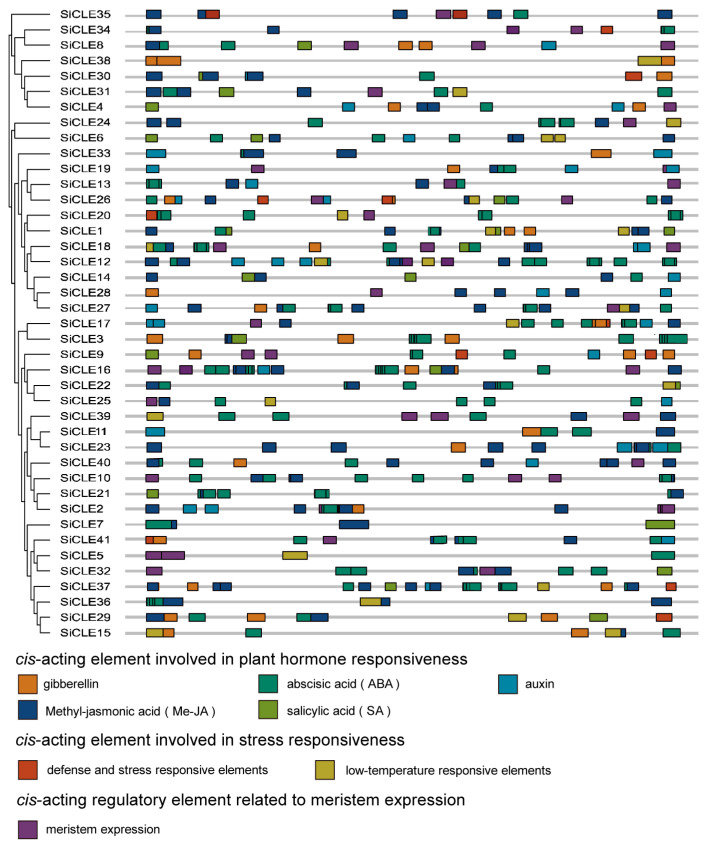
*Cis*-regulatory elements in *SiCLE* gene promoters. The elements are displayed in differently colored boxes.

*Cis*-elements play a crucial role as molecular switches in the transcriptional regulation of genes during plant growth and responses to abiotic stress [56,57,58]. To investigate the potential regulatory mechanisms of *SiCLE* genes in the growth and development of foxtail millet, the 2-kb promoter sequences upstream from the translation start codon of 41 *SiCLEs* were retrieved from foxtail millet genome sequences and analyzed using the PlantCARE website. We identified eight types of *cis*-acting elements grouped into three major categories (Figure 3, Table S3). These elements included plant hormone-responsive elements, including auxin, gibberellin, SA, ABA, and Methyl-jasmonic acid (Me-JA); stress-responsive elements contain low temperature and defense and stress; and elements involved in meristem expression regulation. Notably, multiple types of *cis*-acting elements were unevenly distributed across each *SiCLE* gene. In accordance with previous findings [24], it was observed that each *SiCLE* gene in foxtail millet contained at least one phytohormone-related *cis*-acting element. Most (34/41) *SiCLE* genes possessed more than three types of phytohormone-responsive elements on their promoters, with the exception of *SiCLE5*, *SiCLE10*, *SiCLE20*, *SiCLE24*, *SiCLE36*, *SiCLE38*, and *SiCLE39*. Notably, six *SiCLE* genes (*SiCLE4*, *SiCLE8*, *SiCLE9*, *SiCLE16*, *SiCLE18*, and *SiCLE26*) exhibited all five types of plant hormone-responsive elements, while *SiCLE26* had all eight types of elements. Among the various types of *cis*-acting elements, ABA responsiveness elements were found to be the most abundant in the promoters of *SiCLE* genes in foxtail millet, with an average of 3.66 elements per gene. Notably, *SiCLE16* exhibited the highest number of ABA responsiveness elements, with a total of 11 ABA elements present in its promoter region. Only *SiCLE28* and *SiCLE38* contained no elements involved in ABA responsiveness. On the other hand, the number of *cis*-acting elements involved in defense and stress responsiveness was the lowest, and only nine *SiCLE* genes (*SiCLE9*, *SiCLE17*, *SiCLE20*, *SiCLE26*, *SiCLE29*, *SiCLE30*, *SiCLE34*, *SiCLE37*, and *SiCLE41*) had no more than two elements involved in defense and stress responsiveness. These findings suggest that *SiCLE* genes are not only involved in tissue development processes but also exhibit responsiveness to various plant hormones.

### 3.5. Chromosomal Distribution, Gene Duplication, and Synteny Analysis of the SiCLE Genes

**Figure 4:**
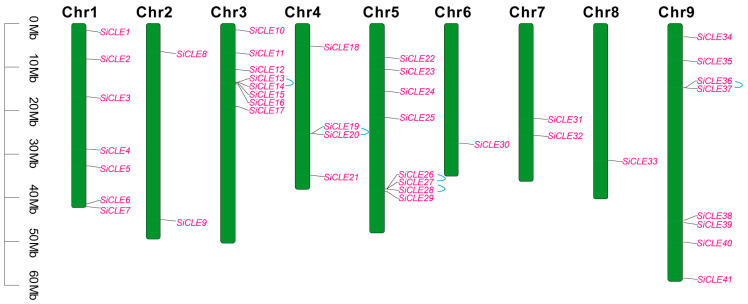
Chromosomal distribution and gene duplication of the *SiCLE* genes. Two *SiCLE* genes of the same segmental duplicated gene pair are labeled with blue lines.

The chromosomal localization analysis of the 41 *SiCLE* genes revealed an uneven distribution across the nine chromosomes of foxtail millet (Figure 4). Among them, Chr3, Chr5, and Chr9 exhibited an equal number of genes, each hosting eight *SiCLE* genes, accounting for 19.51% of the total *SiCLE* genes. Following closely were Chr1 with seven genes (17.07%), Chr4 with four genes (9.76%), and Chr2 and Chr7 with two genes each (4.88%). Chr6 and Chr8 had the least distribution, each containing only one *SiCLE* gene. We then defined tandem duplication events as the presence of two or more genes within a 200 kb chromosomal region [59]. In the *SiCLE* gene family, we identified five tandem duplication events. These included three events on chromosomes Chr3, Chr4, and Chr9, namely *SiCLE13* and *SiCLE14*, *SiCLE19* and *SiCLE20*, and *SiCLE36* and *SiCLE37*, respectively. Additionally, two events were found on Chr5, namely *SiCLE26* and *SiCLE27*, and *SiCLE27* and *SiCLE28*. Among the five pairs of genes with duplication events, four pairs of *SiCLE* genes belonged to Group C, and one pair belonged to Group D. These duplication events are the primary driving force behind the expansion of *SiCLE* genes, and it is possible that Group C and Group D, which possess a relatively large number of *SiCLE* genes, underwent expansion during the whole-genome duplication process. Gene duplication events also led to changes in gene structure. For instance, *SiCLE19* has both 5′UTR and 3′UTR, while *SiCLE20* only possesses a 3′UTR. *SiCLE37* has an additional 3′UTR compared to *SiCLE36*.

**Figure 5:**
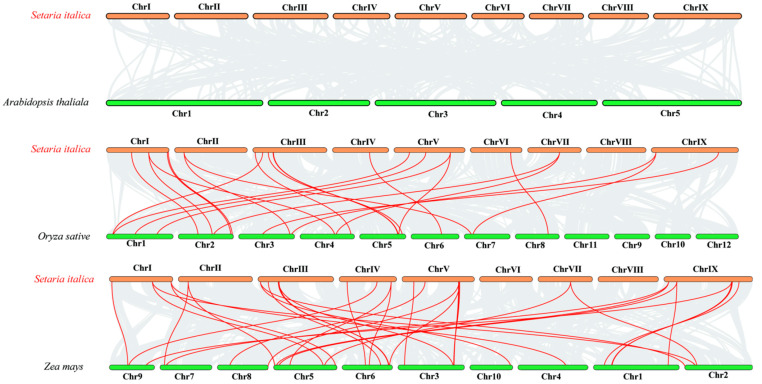
Synteny analyses of the *SiCLE* genes between S. *italica* and three representative plant species. Gray lines on the background indicate the collinear blocks within *S. italica* and other plant genomes; red lines highlight the syntenic *S. italica SiCLE* gene pairs.

To further study the evolutionary mechanism of the *SiCLE* genes in foxtail millet, a collinear map of foxtail millet and three representative species was constructed, including two monocotyledons (rice and maize) and one dicotyledon (Arabidopsis) (Figure 5). As depicted in Figure 5, no collinearity was observed between *CLE* genes in Arabidopsis and foxtail millet, which could be attributed to the distant phylogenetic relationship between dicotyledonous Arabidopsis and monocotyledonous foxtail millet. In monocotyledonous plants, 22 pairs of collinear *CLE* genes were identified between foxtail millet and rice, while the highest number of collinear *CLE* genes was found between foxtail millet and maize, with 30 pairs of collinear *CLE* genes. Notably, the *CLE* genes in maize and foxtail millet exhibited higher collinearity, which may be due to the fact that they are all C_4_ plants.

### 3.6. Expression Pattern Analysis of SiCLE Genes in Different Tissues

**Figure 6:**
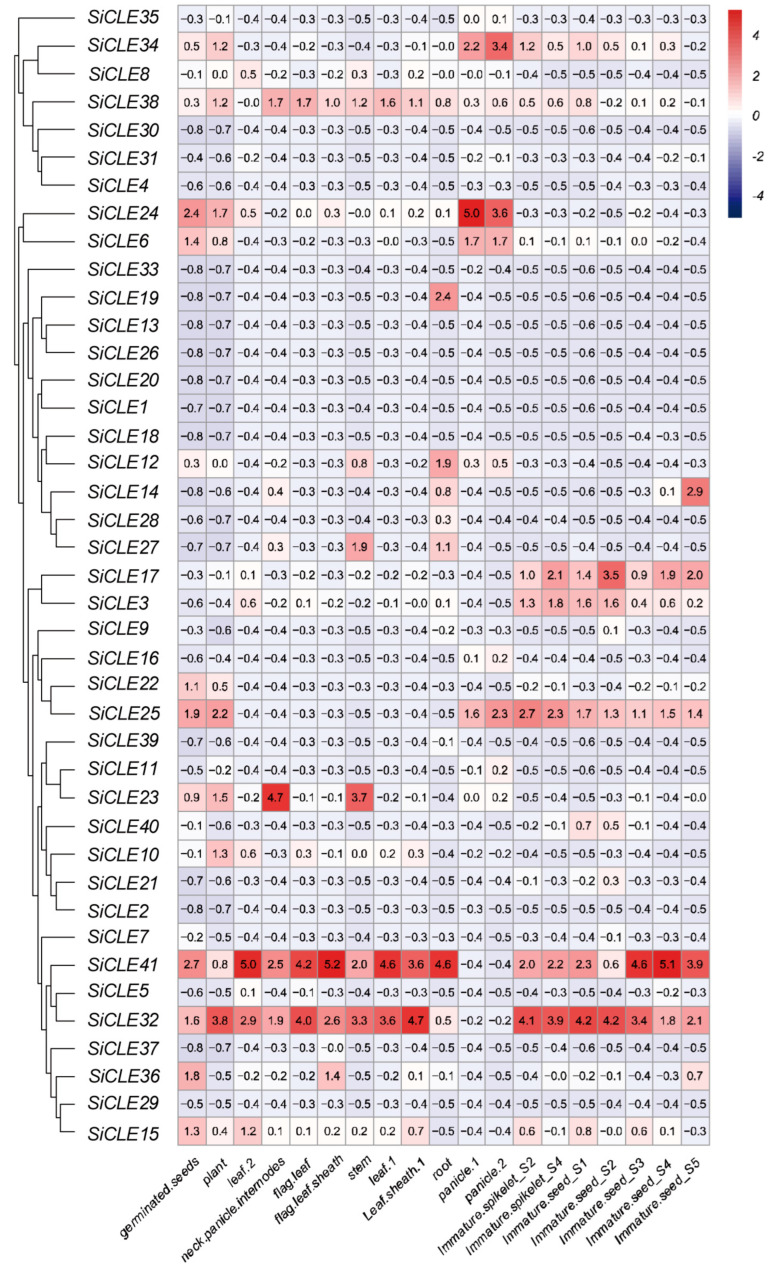
Expression pattern analysis of *SiCLE* genes in different tissues. The heatmap of the expression profiles of the *SiCLE* gene in different developmental stages is represented by normalized values using RNA-seq data, with the color from blue to red indicating the expression levels from high to low.

To comprehensively understand the functions of *SiCLE* genes in foxtail millet, we conducted an analysis of the transcript levels of the 41 *SiCLE* genes in various tissues (root, stem, leaf, panicle, and spikelet) at different growth stages (three days after germination, one-tip-two-leaf stage, heading stage, filling stage, and panicle differentiation stage) in the Jingu 21 variety. We utilized publicly available transcriptome data from MDSi [33] for our analysis. The TPM values of the *SiCLE* genes are listed in Table S4, and a heatmap was generated to display the expression patterns of the *SiCLE* genes (Figure 6). First of all, the expression levels of the majority of *SiCLE* genes remained low throughout the entire period, while a subset of *SiCLE* genes exhibited distinct spatiotemporal specificity. For instance, *SiCLE19* showed higher expression exclusively in roots during the filling stage, whereas *SiCLE14* displayed higher expression in immature seed S5 at the same stage. Additionally, genes clustered within the same group demonstrated similar expression patterns but also exhibited differences. Both *SiCLE32* and *SiCLE41* were classified in Group D, and their expression levels were relatively high in all the tissues except for the panicles. *SiCLE3* and *SiCLE17*, also belonging to Group D, exhibited relatively high expression levels in immature spikelets and immature seeds, while their expression levels in other tissues were low. *SiCLE25*, also classified in Group D, displayed a similar expression pattern to *SiCLE3* but exhibited higher expression in the panicles, germinated seeds, and one-tip-two-leaf plants. *SiCLE23*, another member of Group D, demonstrated high expression in the neck–panicle internodes and stems during the filling stage. Both *SiCLE6* and *SiCLE24* were clustered in Group B, with higher expression levels observed in the panicles, germinated seeds, and one-tip-two-leaf plants, while their expression levels were relatively lower in other tissues. These observations highlight the tissue-specific expression of genes within this family, as well as the similar and specific expression patterns within the same group.

**Figure 7:**
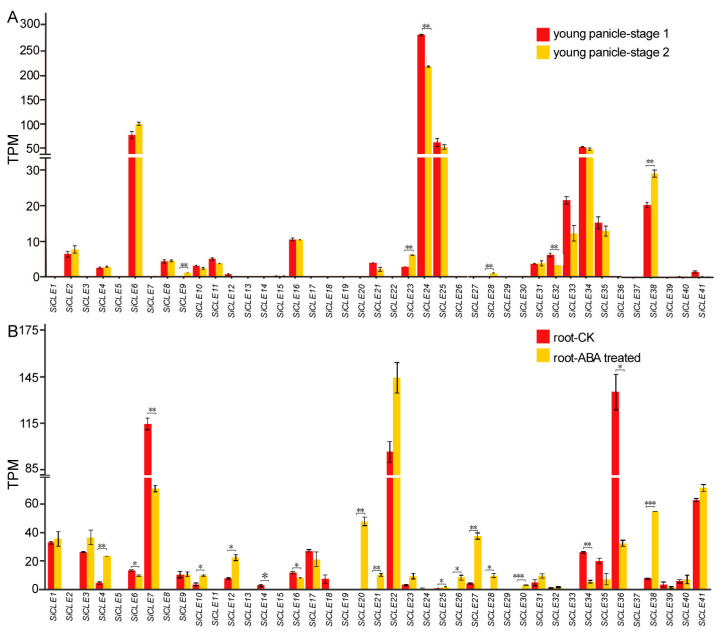
Expression patterns of *SiCLE* genes in young panicles and roots of foxtail millet. (**A**) Expression patterns of *SiCLE* genes in young panicles at different formation stages: branch meristems specified (stage 1, approximately 1.0–1.5 mm) and later stage with clearly formed branch meristems (stage 2, approximately 2.5–3.0 mm). (**B**) Expression patterns of *SiCLE* genes in the roots of 9-day-old seedlings with ABA treatment (2 μM) and without ABA as the control (CK). The bars represent the variation between replicates in the transcriptome sequencing of young panicle samples (**A**) and root samples (**B**). Statistically significant differences between the two stages of the panicle (**A**) and the root with and without ABA treated (**B**) were determined according to *t*-test (* *p* < 0.05, ** *p* < 0.01, *** *p* < 0.001).

CLE peptides play a critical role in regulating stem cell maintenance in plants [60]. Stem cells located within the SAM, RAM, and procambium are established during embryogenesis and continue to generate new tissues and organs during post-embryonic growth and development. The SAM is responsible for the formation of inflorescence meristems, which give rise to flowers and panicles, while the RAM is involved in root growth and development. Transcriptome sequencing was conducted to investigate the expression levels of *SiCLE* genes in young panicles at the formation stage and roots of seedlings (Figure 7). Our analysis revealed that the expression levels of most *SiCLE* genes were relatively low in the young panicle (Figure 7A). Notably, *SiCLE6*, *SiCLE24*, *SiCLE25*, and *SiCLE34* exhibited higher expression levels compared to *SiCLE16*, *SiCLE33*, *SiCLE35*, and *SiCLE38*. Additionally, the remaining *SiCLE* genes showed lower expression levels compared to these eight genes. Based on their expression patterns, these eight genes are included in four groups of the phylogenetic tree: Group A (*SiCLE34*, *SiCLE35*, and *SiCLE38*), Group B (*SiCLE6* and *SiCLE24*), Group C (*SiCLE33*), and Group D (*SiCLE16* and *SiCLE25*). In particular, the expression of *SiCLE24* and *SiCLE32* was significantly reduced in the young panicle at stage 2 compared to stage 1. Conversely, *SiCLE9*, *SiCLE23*, *SiCLE28*, and *SiCLE38* exhibited higher expression in the later stage. Compared to the young panicle, a greater number of *SiCLE* genes exhibited higher expression levels in roots (Figure 7B). Notably, *SiCLE7*, *SiCLE22*, and *SiCLE36* showed the highest expression in roots, followed by *SiCLE41*, *SiCLE1*, *SiCLE17*, *SiCLE3*, *SiCLE34*, and *SiCLE35*, while the remaining genes displayed lower expression levels. These nine *SiCLE* genes are classified into four groups within the phylogenetic tree: Group A (*SiCLE34* and *SiCLE35*), Group C (*SiCLE1*), and Group D (*SiCLE3*, *SiCLE7*, *SiCLE16*, *SiCLE17*, *SiCLE22*, *SiCLE36*, and *SiCLE41*). Moreover, ABA plays a crucial role in regulating root development and growth. It influences root meristem activity, elongation, and branching, thereby modulating overall root architecture [61]. In order to detect the response of *SiCLE* genes after ABA treatment, transcriptome sequencing analysis was performed on the roots of foxtail millet seedlings treated with ABA. The expression of eleven *SiCLE* genes (*SiCLE4*, *SiCLE10*, *SiCLE12*, *SiCLE20*, *SiCLE21*, *SiCLE25*, *SiCLE26*, *SiCLE27*, *SiCLE28*, *SiCLE30*, and *SiCLE38*) was significantly up-regulated, while six *SiCLE* genes (*SiCLE6*, *SiCLE7*, *SiCLE14*, *SiCLE16*, *SiCLE34*, and *SiCLE36*) were significantly down-regulated. The results presented above indicate that the expression of *SiCLE* genes exhibits temporal and spatial specificity, and many members of this gene family are specifically involved in the development of young panicles and roots.

### 3.7. Response of SiCLEs to Plant Hormones in Foxtail Millet

**Figure 8:**
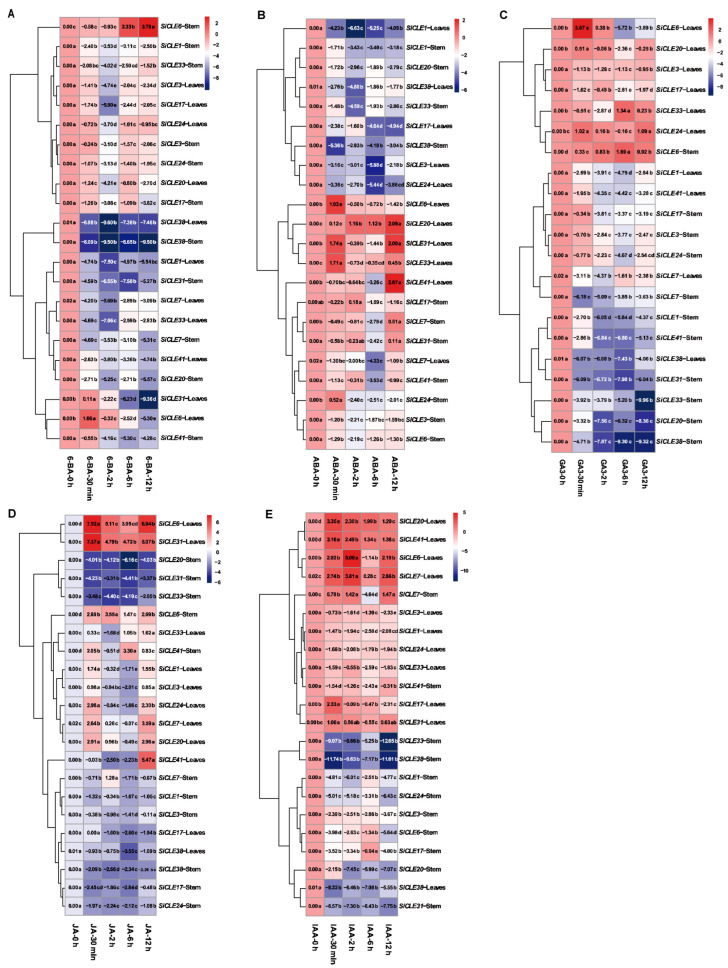
Expression patterns of *SiCLE* genes response to plant hormones in foxtail millet. Seedlings that were 28 days old were treated with plant hormones, including 6-BA (100 µM) (**A**), ABA (100 µM) (**B**), GA3 (100 µM) (**C**), MeJA (100 µM) (**D**), and IAA (100 µM) (**E**). Samples of leaves and stems were collected at 0 h, 0.5 h, 2 h, 6 h, and 12 h after the treatments. The gene expression levels at different time intervals were detected using the qPCR method. The lowercase letters in the heatmap represent significant differences between different time intervals after treatment. The color from red to blue indicates the expression levels from high to low in the heatmap.

The interplay between plant hormones and CLE peptides has been extensively investigated in Arabidopsis, but the understanding of foxtail millet remains limited. To explore the response of *SiCLE* genes to different hormones in foxtail millet, we subjected 28-day-old seedlings to five plant hormone treatments and analyzed the expression patterns of 11 *SiCLE* genes distributed across different groups in leaves and stems (Figure 8). Previous studies have suggested antagonistic effects between cytokinins and CLE peptides in stems and synergistic effects in roots [17]. A similar trend was observed when treating the plants with the cytokinin 6-BA. Except for *SiCLE6*, the expression levels of the other genes were significantly suppressed with prolonged treatment time. Interestingly, within a short duration of 6-BA treatment, these genes exhibited significant changes, with different sensitivities observed among genes in different subgroups. Group C and a subset of Group D genes (*SiCLE3* and *SiCLE17*) showed the lowest expression levels at 2 h of treatment, followed by a gradual recovery. Group A and another subset of Group D genes (*SiCLE7* and *SiCLE41*) maintained a lower expression level even after 2 h of treatment. In contrast, *SiCLE6* from Group B demonstrated clear tissue specificity, with significantly increased expression in stems and a pattern of initial upregulation followed by downregulation in leaves. In ABA treatment, no notable tissue or functional differences were observed among genes in different subgroups. Most genes exhibited their lowest expression levels at 2 or 6 h after treatment, except for *SiCLE6*, *SiCLE20*, *SiCLE31*, and *SiCLE33*, which showed significant upregulation in leaves, potentially associated with stomatal closure [19,62]. Upon short-term application of GA3, with the exception of *SiCLE24*, the other *SiCLE* genes exhibited similar expression patterns across different tissues. Further analysis revealed an antagonistic interaction between *SiCLE* genes and gibberellin during the seedling stage, as their expression was significantly suppressed by GA3, except for *SiCLE6*, which showed increased expression. However, in leaves, the expression of *SiCLE6* remained suppressed with prolonged treatment. JA plays a critical role in plant defense [63]. Treatment of foxtail millet with JA resulted in increased expression of several *SiCLE* genes in leaves within 30 min, including *SiCLE1*, *SiCLE3*, *SiCLE6*, *SiCLE7*, *SiCLE20*, and *SiCLE24*. Notably, *SiCLE6* and *SiCLE31* maintained high expression levels with prolonged treatment. Conversely, in stems, the majority of *SiCLE* genes exhibited downregulation, such as *SiCLE1*, *SiCLE3*, *SiCLE17*, *SiCLE20*, *SiCLE24*, *SiCLE31*, *SiCLE33*, and *SiCLE38*. Hence, there is significant tissue specificity in the response of stems and leaves to JA. Similarly to JA, treatment with IAA caused a significant decrease in the expression of *SiCLE* genes in stems (except for *SiCLE7*), with the strongest inhibitory effect observed on *SiCLE33* and *SiCLE36*. In leaves, some *SiCLE* genes showed upregulation, such as *SiCLE7*, *SiCLE20*, and *SiCLE41*, while others exhibited downregulation, including *SiCLE1*, *SiCLE3*, *SiCLE24*, *SiCLE33*, and *SiCLE38*.

We conducted further analysis to examine the varying response of *SiCLE* genes to different hormones (Figure S3), and notable disparities in *SiCLE* gene expression were observed under different hormone treatments. The majority of *SiCLE* genes exhibited a down-regulation followed by a subsequent recovery in expression levels after treatments with 6-BA, ABA, and GA3. However, certain *SiCLE* genes exhibited exceptions to this trend. For instance, *SiCLE31* in leaves demonstrated sustained downregulation following 6-BA treatment, while *SiCLE6* in stems displayed sustained upregulation after GA3 treatment. In comparison to other hormones, JA and IAA treatments resulted in a higher number of genes exhibiting upregulation at 30 min in leaves. Moreover, the expression patterns of the same genes differed between leaves and stems under identical hormone treatments. For instance, *SiCLE6* showed an upregulation in leaf expression after 30 min of 6-BA treatment, followed by a continuous downregulation as the treatment duration extended. In contrast, in stems, 6-BA treatment initially led to downregulation at 30 min and 2 h, followed by a continuous upregulation with prolonged treatment time. Hence, it can be inferred that different *SiCLE* genes exhibit distinct hormone responses in stems and leaves, suggesting potential functional differences within this gene family across different tissues.

## 4. Discussion

**Figure 9:**
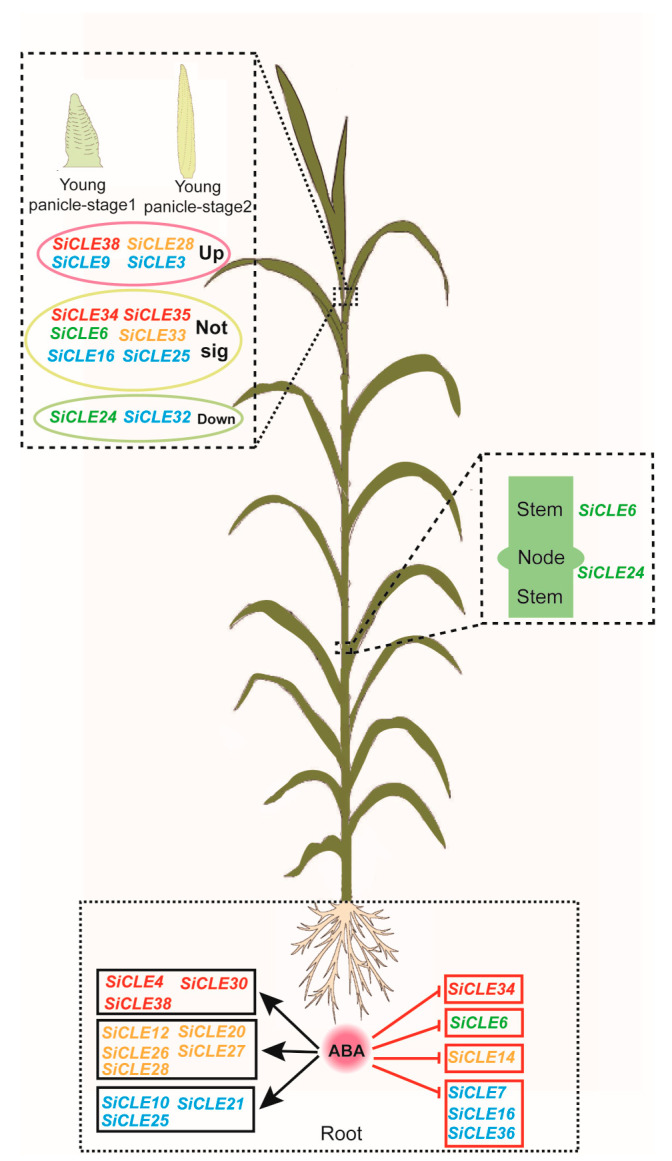
The schematic diagram illustrates the predicted functions of *SiCLE* genes in foxtail millet. The diagram is based on the phylogenetic relationship and expression patterns of *SiCLE* genes, as well as the knowledge from reported *CLE* genes in other species. The font color in the diagram corresponds to the background color of different groups in the phylogenetic tree.

Intercellular communication is crucial for coordinating growth and development in eukaryotes. In plants, various signaling molecules such as plant hormones, polypeptides, and small RNAs transmit information between cells [1,64,65]. Polypeptides, in particular, play a significant role in cell signaling within plants [66]. Many small peptides act as secretory signaling molecules that are sensed by specific receptor kinases located on the cell membrane. This interaction mediates complex intercellular communication and induces responses from neighboring cells, including the regulation of meristem homeostasis [67,68]. The *CLE* gene family represents one of the largest known polypeptide families in plants. These genes are present throughout the evolutionary lineage of terrestrial plants [36,69,70] and generally play a conserved role in regulating plant development and physiology [71,72]. Consistent with the findings of this study, the expression patterns of *SiCLE* genes exhibited distinct spatiotemporal specificity (Figure 6), indicating their diverse functions in plant development. For instance, during the filling stage, *SiCLE19* exhibited higher expression exclusively in roots, while *SiCLE14* displayed higher expression in immature seed S5. Within the same gene groups, *SiCLE* genes showed similar but distinct expression patterns. In Group B, *SiCLE6* and *SiCLE24* demonstrated a similar expression pattern, with higher expression levels observed in panicles, germinated seeds, and one-tip-two-leaf plants while exhibiting relatively lower expression levels in other tissues. Similarly, in Group D, *SiCLE32* and *SiCLE41* showed relatively high expression levels in all tissues except for the panicles. On the other hand, members such as *SiCLE3* and *SiCLE17* in Group D displayed relatively high expression levels in immature spikelets and immature seeds. Furthermore, transcriptome sequencing was conducted to investigate the expression levels of *SiCLE* genes in young panicles during the formation stage and in seedling roots (Figure 7). Most *SiCLE* genes exhibited relatively low expression levels in young panicles. *SiCLE6*, *SiCLE24*, *SiCLE25*, and *SiCLE34* showed higher expression levels in young panicles, with *SiCLE24* showing a significant reduction in expression during the later stage 2 of panicle development. In comparison, a greater number of *SiCLE* genes exhibited higher expression levels in roots. *SiCLE7*, *SiCLE22*, and *SiCLE36* displayed the highest expression levels in roots, and the expression of *SiCLE7 and SiCLE36* was significantly down-regulated after ABA treatment. Our study indicates that the expression of *SiCLE* genes demonstrates temporal and spatial specificity, with many members of this gene family playing specific roles in the development of young panicles and roots. The predicted functions of *SiCLE* genes in foxtail millet are shown in Figure 9.

Numerous *CLE* loci have been identified across different species [69], and a recent study uncovered 104 *CLE* loci in bread wheat [28]. In the case of foxtail millet, a total of 41 *SiCLE* genes were identified in our study (Table 1). All the *SiCLEs* were distributed on nine chromosomes and divided into four groups, Group A-D (Figure 1). Five pairs of genes with duplication events, four pairs of *SiCLE* genes belonged to Group C, and one pair belonged to Group D (Figure 4). *SiCLE* genes within the same phylogenetic group exhibited similar features and displayed a diverse gene structure (Figure 2). Although the overall sequence conservation of SiCLE peptides is limited, they have a molecular weight of less than 15 kDa, and most of the polypeptides are highly basic and possess signal peptides at the N-terminal. This molecular characteristic is highly similar to CLE polypeptides found in other species [48,72]. Although these *SiCLE* genes exhibit diverse structural features, they all possess a conserved CLE domain located at the C-terminal, which is crucial for the functional specificity of the CLE family [73]. In species like rice, wheat, and *Medicago truncatula*, genes producing multiple conserved CLE domains have been found due to series repetition [36]. This structural arrangement promotes the generation of more effective CLE peptides [74]. However, such a phenomenon was not observed in foxtail millet, suggesting that the *SiCLE* genes in foxtail millet are highly conserved and have undergone minimal changes throughout evolution. Each *CLE* gene encodes a pre-propeptide consisting of a signal sequence that guides the protein through the secretory pathway, a central variable region, and a highly conserved CLE domain located at the C-terminal [71]. The full-length propeptide undergoes proteolysis [75], resulting in the production of a mature polypeptide comprising 12–14 amino acids from the CLE domain. This mature polypeptide can undergo additional post-translational modifications [14,76,77]. In our study, five conserved motifs were identified (Figure 2). Motif 1 contained the typical CLE domain and was located at the C-terminus of all 41 SiCLE peptides. Motif 2 was found in the majority of SiCLE peptides and located near the N-terminus. Motifs 3, 4, and 5 were in the central variable region, and only a few SiCLE peptides contained them. The comparative analysis of conserved CLE sequences revealed a high degree of similarity between foxtail millet and the other three species, including Arabidopsis, rice, and maize (Figure S2). The collinearity of foxtail millet and the three species indicates that *CLE* genes in maize and foxtail millet exhibited higher collinearity with 30 collinear gene pairs, which may be due to the fact that they are all C_4_ plants.

The *CLE* gene family in Arabidopsis is classified into type A and type B based on their functional characteristics, specifically whether they participate in the differentiation of root or stem meristem cells [78]. Type A CLE peptides include CLV3, CLE1-CLE27, CLE40, and CLE45. Based on clustering analysis, it is evident that most A-type CLE peptides are grouped in clusters C and D, indicating their important roles in the differentiation of plant root or stem meristem cells. On the other hand, B-type CLE peptides are clustered in Groups A and B, suggesting their potential involvement in plant vascular bundle development [79]. The homeostasis of plant stem cells is primarily maintained through a negative feedback pathway involving *WUS* and *CLV3*. Genes such as *AtCLV3*, *OsFON2/FON4*, *OsFCP1*, *OsFCP2*, *ZmCLE7*, *ZmFCP1*, and others have been extensively studied and demonstrated to be essential members of this pathway in their respective species [10,11,49,50]. These genes have been found to play crucial roles in extracellular signaling for meristem maintenance. In addition, the CLV3 homolog in other species also significantly influences the regulation of floral meristem size, the number of floral organs, and even the fruit size, such as *Setaria viridis* [80], cucumber [81], tomato [82], and *B. napus* [83,84]. The pre-propeptides encoded by these genes are all clustered in Group C (Figure 1), suggesting that the *SiCLE* genes clustered in Group C may also have similar functions in regulating floral meristem size and the number of floral organs in foxtail millet. Notably, we observed that the gene *SiCLE33*, located in Group C, exhibited higher expression levels during the transcriptome sequencing of the young panicle development stage. This indicates that *SiCLE33* may play a vital role in CLV-WUS feedback signal transduction in foxtail millet. Furthermore, we found that genes clustered within the same group shared not only similar gene structures and relatively conserved motif compositions (Figure 2) but also displayed similar expression patterns of *SiCLE* genes throughout the entire life cycle of foxtail millet, as revealed by transcriptome data (Figure 6 and Figure 7). These findings are significant in comprehending the functions of SiCLE peptides in foxtail millet. Mature CLE peptides function by binding to membrane-bound receptors from various families, thereby transmitting CLE signals into cells and regulating downstream transcription factors and plant hormone signaling pathways [1]. The interaction between phytohormone signaling and CLE peptides has been extensively studied in Arabidopsis. For example, *AtCLE42* can delay leaf senescence by suppressing ethylene biosynthesis [16], while *AtCLE6* plays a systemic role in shoot growth under the influence of gibberellin in Arabidopsis [18]. *AtCLE5* and *AtCLE6* are specifically expressed at the base of developing leaves and floral organs, with their transcript levels regulated by auxin to modulate final leaf morphology [20]. In our study, we identified eight types of *cis*-acting elements in the upstream 2-kb region of the 41 *SiCLE* genes (Figure 3, Table S3). Five of these elements were related to plant hormone response, including auxin, GA3, SA, ABA, and Me-JA responsive elements. These findings suggest possible interactions between SiCLE genes and this phytohormone signaling in foxtail millet. The frequent occurrence of ABA and Me-JA *cis*-acting elements indicates their significant roles in regulating the expression of *SiCLE* genes. To investigate further, we treated foxtail millet seedlings with five different hormones during the early growth stage and observed that the expression of the majority of *SiCLE* genes was downregulated under different hormone treatments (Figure 8 and Figure S3). Interestingly, even under the same hormone treatment, different SiCLE genes displayed distinct expression patterns in leaves and stems, implying potential functional differences within this gene family across different tissues. The roles of AtCLE peptides have been well established in a range of developmental and physiological processes, including shoot stem cell homeostasis, root xylem development, root protophloem cell differentiation, vascular cambium activity, and stomatal formation and closure [1]. As the functions of AtCLE peptide signaling pathways have been extensively elucidated in *A. thaliana*, our study will contribute to exploring the diverse functions of SiCLE peptides in various developmental and physiological processes in foxtail millet.

## 5. Conclusions

The present study provides the first comprehensive identification and analysis of the *SiCLE* gene family in foxtail millet. A total of 41 *SiCLE* genes were distributed on nine chromosomes, with five pairs of gene duplication events, which were divided into four groups. *SiCLE* genes within the same phylogenetic group exhibited comparable gene structure and motif patterns. All the *SiCLE* proteins had the C-terminal conserved CLE domain and highly conserved positions of CLE core sequences between foxtail millet, Arabidopsis, rice, and maize. The *SiCLE* genes had eight types of cis-elements, and five of them were plant hormone-responsive elements, including auxin, gibberellin, salicylic acid, ABA, and Me-JA. Thirty-four *SiCLE* genes possessed more than three types of phytohormone-responsive elements on their promoters. *CLE* genes in maize and foxtail millet exhibited higher collinearity with 30 pairs of collinear *CLE* genes, which may be due to the fact that they are all C_4_ plants. The tissue-specific expression patterns and plant hormone responsiveness of *SiCLE* genes suggest their crucial involvement in various aspects of plant development and physiology. These findings provide a foundation for further in-depth investigations into the functional characterization and evolutionary aspects of *SiCLE* genes in foxtail millet.

## Figures and Tables

**Table 1:** The properties of *SiCLE* genes.

Gene ID	Gene Name	Chromosome Location	Signal Peptide Prediction	Number of Amino Acids	Molecular Weight (kD)	Isoelectric Poin	GRAVY
*Si1g02740*	*SiCLE1*	Chr1:1701179–1702028	+	83	8.67	5.45	0.071
*Si1g09260*	*SiCLE2*	Chr1:8075455–8076779	+	110	10.98	10.87	−0.06
*Si1g15040*	*SiCLE3*	Chr1:16870079–16870441	+	120	13.42	11.20	−0.497
*Si1g21450*	*SiCLE4*	Chr1:28806486–28807552	+	91	9.66	10.39	−0.204
*Si1g25600*	*SiCLE5*	Chr1:32729889–32731715	+	85	8.68	9.49	0.232
*Si1g36220*	*SiCLE6*	Chr1:41284255–41285566	−	98	10.46	11.83	−0.427
*Si1g37440*	*SiCLE7*	Chr1:42036658–42038549	+	85	9.70	9.99	−0.359
*Si2g07840*	*SiCLE8*	Chr2:6541423–6542683	−	104	10.92	11.49	−0.34
*Si2g37520*	*SiCLE9*	Chr2:45004975–45005241	+	88	9.12	6.03	−0.016
*Si3g03240*	*SiCLE10*	Chr3:1462866–1464336	+	92	9.50	11.01	−0.049
*Si3g10460*	*SiCLE11*	Chr3:6732083–6733514	+	86	9.26	11.83	−0.2
*Si3g14760*	*SiCLE12*	Chr3:10562885–10563172	+	95	9.64	8.25	0.082
*Si3g17810*	*SiCLE13*	Chr3:13430959–13431192	+	77	8.02	9.18	0.112
*Si3g17830*	*SiCLE14*	Chr3:13472475–13472753	+	92	9.27	5.68	0
*Si3g18030*	*SiCLE15*	Chr3:13627896–13628249	+	117	12.66	11.25	−0.368
*Si3g18460*	*SiCLE16*	Chr3:13936956–13938953	−	99	10.24	11.34	−0.269
*Si3g22990*	*SiCLE17*	Chr3:19091857–19092216	+	119	13.17	11.50	−0.403
*Si4g07460*	*SiCLE18*	Chr4:5221231–5221955	+	83	8.54	7.78	0.094
*Si4g16140*	*SiCLE19*	Chr4:25203756–25204438	+	119	12.22	9.98	−0.317
*Si4g16170*	*SiCLE20*	Chr4:25243962–25244464	+	82	8.56	5.98	0.126
*Si4g23620*	*SiCLE21*	Chr4:34831659–34833580	+	127	12.86	11.03	−0.366
*Si5g09450*	*SiCLE22*	Chr5:7782941–7783884	+	93	9.72	11.86	0.103
*Si5g12400*	*SiCLE23*	Chr5:10508454–10508813	+	119	12.50	11.89	−0.3
*Si5g16690*	*SiCLE24*	Chr5:15497255–15497578	+	107	10.95	11.91	0.068
*Si5g18600*	*SiCLE25*	Chr5:21570492–21570803	+	103	11.00	12.16	−0.194
*Si5g32640*	*SiCLE26*	Chr5:37962048–37962314	+	88	8.93	11.65	−0.086
*Si5g32660*	*SiCLE27*	Chr5:37979626–37979868	+	80	7.93	6.57	0.173
*Si5g32670*	*SiCLE28*	Chr5:37981960–37982229	+	89	9.14	9.10	−0.128
*Si5g33310*	*SiCLE29*	Chr5:38510416–38510766	+	116	12.87	9.75	−0.422
*Si6g16350*	*SiCLE30*	Chr6:27510936–27511193	+	85	9.61	11.42	−0.351
*Si7g13900*	*SiCLE31*	Chr7:21729033–21729898	+	113	11.40	11.66	−0.113
*Si7g19100*	*SiCLE32*	Chr7:25632473–25632760	+	95	10.07	10.74	−0.065
*Si8g17400*	*SiCLE33*	Chr8:31420418–31420996	+	100	10.33	8.97	−0.068
*Si9g05550*	*SiCLE34*	Chr9:3007939–3009361	−	99	10.28	11.34	−0.362
*Si9g13150*	*SiCLE35*	Chr9:8512203–8514840	+	103	10.46	10.24	−0.087
*Si9g19660*	*SiCLE36*	Chr9:14748515–14748763	+	82	9.10	6.56	−0.402
*Si9g19670*	*SiCLE37*	Chr9:14751454–14751981	+	101	11.09	10.22	0.02
*Si9g38030*	*SiCLE38*	Chr9:45088642–45088950	+	102	10.44	11.11	0.047
*Si9g38660*	*SiCLE39*	Chr9:45679290–45679866	+	94	10.38	9.81	−0.068
*Si9g44430*	*SiCLE40*	Chr9:50178960–50179208	−	82	8.53	11.61	0.263
*Si9g55410*	*SiCLE41*	Chr9:58385979–58386266	+	95	10.84	10.00	−0.287

## Data Availability

All data generated or analyzed during this study are included in this published article.

## Supplementary Materials

The following supporting information can be downloaded at https://www.mdpi.com/article/10.3390/genes14112046/s1. Figure S1: Weblogo plots of the 5 conserved motifs of SiCLE peptides; Figure S2: A comparative analysis of CLE core sequences among foxtail millet, Arabidopsis, rice, and maize; Figure S3: Response of *SiCLE* genes to different plant hormones; Table S1: Primers used to amplify the *SiCLE* genes and reference gene for qPCR; Table S2: *CLE* genes used for phylogenetic tree in Arabidopsis, rice, and maize; Table S3: Major *cis*-regulatory elements in *SiCLE* gene promoters. Table S4: The Transcripts Per Million (TPM) values of the *SiCLE* genes in different tissues.
