# Supplementary material for: Genome-Wide Identification and Characterization of CLAVATA3/EMBRYO SURROUNDING REGION (CLE) Gene Family in Foxtail Millet (Setaria italica L.)
Source: Genes (Basel). 2023 Nov 6;14(11):2046. doi: 10.3390/genes14112046 (PMC10671770; doi:10.3390/genes14112046)
Supplement: Supplementary file 1 [file genes-14-02046-s001.zip › Supplementary Figure legends.pdf]

Figure S1. Weblogo plots of the 5 conserved motifs of SiCLE peptids.

Figure S2. A comparative analysis of CLE core sequences among foxtail millet, Arabidopsis, rice, and maize.

Figure S3. Response of *SiCLE* genes to different plant hormones. Different plant hormones are labeled in different colour.
